# The impact of green bond issuance on carbon emission intensity and path analysis

**DOI:** 10.1371/journal.pone.0304364

**Published:** 2024-06-05

**Authors:** Haifeng Pang, Changxu Wu, Liucheng Zhang

**Affiliations:** 1 Harbin Business University, School of Finance, Harbin, Heilongjiang Province, China; 2 Harbin Business University, Harbin, Heilongjiang Province, China; National University of Sciences and Technology, PAKISTAN

## Abstract

Reducing carbon emission intensity is crucial for achieving sustainable development. Carbon emission intensity is expressively affected by the issuance of green bonds. Thus, it is imperative to assess the influence of green bond issuance on carbon emissions and examine their correlation. Such research holds great potential to expedite the overhaul and modernization of businesses and to construct a circular economy system. This paper uses the spatial Durbin model to draw empirical conclusions by using data from 26 provinces in China between 2016 and 2021. Firstly, under different spatial matrices, it has been analyzed that an increase of 1% in the issuance of green bonds leads to a reduction of 0.306% or 0.331% in carbon emission intensity. It shows that green bonds have the potential to substantially reduce carbon intensity. Additionally, the intensity of emissions in the current period is driven by the intensity of emissions in the previous period. Secondly, the analysis of mediated transmission suggests that green bonds can ultimately reduce carbon emission intensity by changing the energy consumption structure or improving the efficiency of green technology innovation. Thirdly, the analysis of heterogeneity shows that the inhibitory effect of green bond issuance on carbon emissions is stronger in less economically developed regions than in economically developed regions. There is a significant inhibitory effect of green bond issuance in neighboring provinces on local carbon emission intensity. This effect is present only in provinces in less economically developed regions and not in economically developed regions.

## 1. Introduction and literature review

As we enter the twenty-first century, it is increasingly evident that excessive carbon emissions cause harm. Greenhouse gases, if emitted excessively, will have irreversible negative impacts on human daily life and the global ecological environment. The 2005 Protocol’s formal entry into force demonstrates that reducing carbon emissions has become a major goal for all countries in their pursuit of sustainable development and global economic growth. Efficiently dealing with the pressure caused by carbon emissions has become a major social issue for China, a large carbon-emitting country. This issue not only promotes the country’s high-quality development but is also key to China’s successful transformation in the new era. China, as a major carbon-emitting country, has taken the initiative to reduce carbon emissions. In October 2021, China released the white paper ’China’s Policies and Actions to Address Climate Change,’ which highlights the country’s commitment to addressing climate change. The paper outlines measures and programs to reduce the intensity of carbon emissions and promote green economic and social development.

Scholars have approached the study of emission reduction from various perspectives. Currently, there is no consensus among scholars regarding the effect of financial development. During the initial stages of research and analysis, it was discovered that an increase in financial development leads to an expansion in production scale, which exacerbates carbon emissions (Dasgupta et al., 2001 [1]). However, Itit has been suggested by scholars that an increase in financial development is associated with a decrease in per capita carbon emissions (Shahbaz et al., 2013 [2]). Despite the development and changes over time, disagreement still exists. Among the various views on the relationship between the development of the financial sector and carbon emissions, the opinion that financial development inhibits carbon emissions is supported by a majority of scholars (Shao and Liu., 2017 [3]; Shahbaz et al., 2018 [4]; Muzzammil et al., 2022 [5]). However, a minority of scholars remain skeptical. Salahuddin et al. (2015) [6] conducted empirical research and found that financial development accelerates economic growth and increases energy consumption, thereby exacerbating the emissions of carbon. Dogan and Seker (2016) [7], Yan et al. (2016) [8] and Huang et al. (2021) [9] conducted research and the non-linear relationship between financial development and carbon emissions was found. Secondly, on the impact of green finance, researchers have not reached a unanimous conclusion. The majority of researchers are in agreement that green finance has a positive impact on the reduction of carbon emissions, Jiang et al. (2020) [10] and Arshian et al. (2022) [11] analyze the data based on the samples of 23 provinces in China and the Group of Seven, respectively, and find that green finance plays an obvious inhibitory effect on carbon emissions. There are also a few scholars who hold a negative view: Wan and Sheng (2021) [12] and Cao et al. (2021) [13] empirically analyze that it is not yet clear whether green finance will have a significant impact on the carbon emissions of the economy through the use of the linked equation model and the double difference method, respectively. In addition, there are scholars who discuss and analyze the influencing factors from other multiple perspectives and draw corresponding conclusions. For example, Sun et al. (2023) [14] combined the spatial Durbin model and the threshold regression model to analyze that the carbon productivity spatially presents high (low)-high (low) point-like spatial clustering characteristics; Guo and Sun (2017) [15], Anser et al. (2020) [16] take China and the Union of South Asian Nations as the background, respectively, using the LMDI model and the STIRPAT model to study and analyze that the population size has a positive role in promoting carbon emissions; Zhang and Zhang (2022) [17] analyze that environmental regulation is conducive to guiding enterprises to optimize in the direction of high-energy and low-pollution, so as to achieve the purpose of reducing carbon emissions. Meanwhile, direct investment (Qu and Luo, 2021 [18]), trade exchanges (Zhang et al., 2017 [19]), the level of urbanization (Zhang et al., 2016 [20]; Qi and Guo, 2022 [21]), financial and environmental protection expenditure (Li and Huang, 2022 [22]) are also studied and discussed by scholars. Finally, there have been extensive studies on the emission effect. As China entered a new development mode, the industrial CO_2_ emissions of the Chinese economy were analysed (Song et al., 2021 [23]). They used the Tapio model and logarithmic mean index of differentiation to conclude that industrial CO_2_ emissions shifted from high levels in the northern coastal regions to lower levels in the rest of China. The main inhibiting factors identified by the study in different periods were energy intensity and industrial structure. The distribution of industrial CO_2_ emissions has shifted over time, with higher emissions now occurring in the northern coastal areas and lower emissions in the southern areas. Further analysis indicates that energy intensity and industrial structure were the primary factors inhibiting emissions in different periods. Liu et al. (2023) [24] analyzed CO_2_ emissions from agricultural land in China between 1995 and 2020 using the Tapio decoupling model and the log-mean divergence index. They found that films and agriculture are the primary sources of anthropogenic CO_2_ emissions from agricultural land. Further analysis reveals that the level of agricultural economy is a decisive factor in promoting the increase of CO_2_ emissions. Jia et al. (2023) [25] analyzed the overall increasing trend of carbon emissions from tourism through a combination of social network analysis and the log-mean Divisia index. They concluded that the scale of tourists is the main driving force, while energy intensity is the inhibitor factor of greatest significance.

Green bonds are a crucial element of the green financial system. They can effectively direct funds towards green fields, solve the issue of insufficient funding for green low-carbon projects, and promote the gradual transformation of enterprises towards green and low-carbon practices. Additionally, they can contribute to the overall development of society. The market scale of green bonds is gradually expanding with the introduction of green bond principles and climate bond organizations in the international market. In 2015, the Hong Kong Stock Exchange issued the first Chinese green bonds, marking the official start of China’s green securities market development journey. By the end of 2022, it is expected that China’s domestic and foreign green bond stock will reach approximately 3 trillion yuan, with a new issuance size of around 983.899 billion yuan. The global green bond market reached a record high in total issuance despite the impact of the COVID-19 pandemic in 2020. However, in the first half of 2022, the total number of new green bonds issued was 21% lower than the same period in 2021 due to inflationary concerns and market turmoil caused by the international conflict between Russia and Ukraine. Nowadays, the world is facing a problem with limited natural resources and ecological damage. All countries are seeking new ways of economic growth and development. Green bonds are a promising financing option worth exploring. Scholars have mainly researched this topic from two aspects, as seen in existing literature.

On the one hand, issues regarding the pricing of green bond issuances, return risk, and financing costs are present. Firstly, scholars both domestically and abroad have analyzed the "green premium" effect of green bonds, but no unanimous conclusion has been reached. Several scholars have discovered proof of the "green premium" exist through their analysis of comparative regular claims, despite the varying degrees of discounting in different regions (Britta and Dirk, 2018 [26]; Zerbib, 2018 [27]; Wang et al., 2020 [28]). Some researchers further investigate and determine that the premium is contingent. Consequently, there is a substantial green premium only if the issuer is a lower credit risk entity (Kapraun and Scheins, 2019 [29]), or if the issuance has received authoritative green certification (Hyun et al., 2020 [30]). Similarly, some scholars have presented an opposing view. Ehlers and Packer (2017) [31] found that despite a significant premium upon issuance, ordinary debentures and green bonds perform similarly in trading on the secondary market. In a similar vein, Larcker and Watts (2020) [32] concluded that there are minimal differences between green and non-green bonds across various aspects, making them nearly identical substitutes. Furthermore, utilizing the fixed effects model (Malcolm et al., 2022 [33]; Sun and Lei, 2023 [34]) and the feasible generalized least squares model (Andersson and Prag, 2015 [35]), scholars have comprehensively analyzed the green bond markets in the United States and Sweden, they found green bonds to be priced significantly higher than traditional bonds. Secondly, scholars have also discussed and researched the factors that influence the issue price and financing costs of green bonds, which are highly valued topics. Scholars have determined through econometric modeling and empirical analysis that various factors influence the issuance price of green bonds. Among these factors are the adjustment of third-party certification (Karpf and Mandel, 2018 [36]) and so on. Although the effect on liquidity risk remains less evident (Febi et al., 2018 [37]). Concerning financing cost influencing factors, empirical analysis research has concluded that higher liquidity (Zerbib, 2018 [27]), certification by a trust institution (Eichholtz et al., 2019 [38]), and a higher ESG rating of the company (Antonio and Ana, 2021 [39]) result in lower financing costs for green bonds.

On the other hand, this pertains to the economic outcomes of green bonds. In terms of the study content, scholars have initiated discussions on the economic impact of green bonds. One main finding is that green bonds enhance energy conservation and reduce emissions positively. Some scholars posit that the implementation of a green financial system can drive capital towards environmentally friendly and low-emission industries by influencing financing costs and availability. This supports the notion that green bonds could potentially aid in reducing carbon emissions (Jun, 2015 [40]; Wang et al., 2023 [41]). Ning and Wang (2021) [42] analyzed green bonds’ impact on corporate financing costs and constraints can be examined through the lenses of maturity mismatch and investor sentiment. The results of their analysis confirmed that green bonds can effectively reduce the cost of financing and the need of fund, helping businesses transform and grow. Additionally, this research examines the effect of green bonds on the stock price of companies. Before the issuance of green bonds, numerous scholars both domestically and internationally have conducted studies on the effect of ordinary debenture issuance on a company’s stock price. Findings have shown either a negative impact (Clifford et al. and SMITH, 1986 [43]; Liu, 2005 [44]; Fu et al., 2010 [45]) or no significant impact (Castillo, 2004 [46]). Scholars have focused on studying green bonds issuance. Based on empirical analysis of relevant data from six countries, Roslen et al. (2017) [47] concluded that green bonds have no impact on all events. Meanwhile, Chen (2018) [48], Liang (2018) [49], Chen and Zhang (2022) [50] conducted a study analyzing the stock prices of green bond market in China to enhance the persuasiveness of their conclusion.

This paper focuses on green bond issuance and carbon intensity in the country with the highest energy consumption and carbon emissions in the world. The research is conducted on 26 provincial administrative regions in China. Since China established the green bond system at the end of 2015, the latest data from 2016 to 2021 is analyzed to determine the impact of green bond issuance on carbon emissions. This study analyzes the transmission mechanism of green bonds on carbon emissions by considering the intermediary transmission effect. Based on the empirical analysis results, corresponding suggestions are proposed. These suggestions can guide the transformation of the market structure and facilitate the harmonious development of human society and the ecological environment.

## 2. Theoretical analysis

Issuing Green Bonds directly impacts carbon emissions by combining market-based environmental regulation with resource allocation. Unlike current command and incentive regulation, green bonds efficiently allocate limited financial resources to optimize the balance between the economic and environmental sectors, utilizing their internal core features.

The bond issue aims to offer financial support to the market while providing directed financial assistance to the green industry. Additionally, it aims to define the field and scope in a unified manner to facilitate accurate support for the environmental transition in China. The bond issue will play a key role in developing critical projects by providing corresponding financial funding. Environmental factors have prompted financial institutions to integrate green project and enterprise credit and project management into their system. This allows for targeted financial support for green, low-carbon projects and enterprises, while reducing funds for high-pollution and high-impact ventures. Overall, this promotes internal green transformation. Second, green bonds can also lower the expenses of environmentally friendly financing for businesses. The process of green enterprise transformation differs from basic enterprise transformation and involves uncertain outcomes and extended profit cycles, which impose significant financing constraints on enterprises investing in green projects. However, issuers of green bonds are more preferred by investors due to the benefit of lower credit spreads (Qi and Liu, 2021 [51]), thus securing funds at lower costs (Wu et al., 2022 [52]), and the People’s Bank of China designated green bonds as qualified collaterals in their open market operations, this has led to a rise in the issuance price and a decrease in the financing cost, thereby encourage corporate participation in environmental management. Thirdly, green bond issuance can enhance issuer information transparency. The conventional bond market lacks oversight over enterprises’ environmental conduct and investment flow, precluding notable advantages. The "Green Bond Assessment and Certification Organization Market-based Review Operating Rules (for Trial Implementation)" and other supporting documents can encourage third-party professional organizations to assess the utilization of accrued funds and improving the caliber of certification for green bonds. This, in turn, can prevent the occurrence of "greenwash" behavior and ensure the full utilization of funds for the relevant green projects. Green Project Construction. This paper proposes hypothesis 1 based on the analysis.

Hypothesis 1: Green bond issuance assists in reducing carbon emissions within the region.

The quality of the environment is significantly affected by changes in the structure of energy consumption. For example, a high proportion of fossil fuel consumption leads to increased carbon dioxide emissions. Upgrading and optimizing the energy structure effectively reduces carbon emissions. Green bonds were analyzed from the perspectives of production and consumption. From a consumption standpoint, green bonds can stimulate green development consciousness in enterprises through price competition, encourage low-carbon green upgrading, and facilitate the transition to sustainable consumption structures. On the production side, Liu and Ren (2019) [53] found that green finance can achieve the objective of promoting relevant enterprise equipment and technology while reducing carbon emissions by converting environmental pollution into enterprise financing costs. Achieving the promotion of upgraded technology for equipment within the enterprise is a major aspect of green finance. Green bonds also contribute to this goal by increasing the use of clean energy and decreasing reliance on fossil fuels through continuous green upgrades. This leads to improvements in the structure of energy consumption and ultimately helps to carbon emission reduction. Green bonds serve as a means of capital diversion, utilizing policy tools to guide funds towards green industries, while simultaneously advancing the development of green, low-carbon industries and increasing the production and consumption of clean energy. Through these efforts, the reduction of emissions is achieved. Hypothesis 2 is presented based on the aforementioned analysis.

Hypothesis 2: By changing the structure of energy consumption, green bonds achieve carbon emission reduction.

Green bonds can boost the advancements of green science by promoting their efficiency, which in turn significantly reduces carbon emissions. Green technology, being relatively new, has the potential to utilize the ecological environment more efficiently with its own energy-saving and carbon reduction capabilities. However, the innovation of green technology itself entails high-risk, high investment, and long cycles, leading to inconsistent preferences in the collateral process of bank credit. This generates credit constraints, affecting the circulation of the capital chain, coupled with the high cost of complying with environmental regulations, resulting in a "crowding out" effect. Under these conditions, green bonds can help ease the burden of financing and optimize the internal debt structure of businesses due to their advantages. This will promote a shift towards green, low-carbon, environmentally friendly and efficient production modes supported by the influence of various factors, leading to a reduction in carbon emissions. In this process, the spillover effect can accelerate the dissemination and green technology application, leading to a reduction in carbon emissions. Hypothesis 3 is presented based on the aforementioned analysis.

Hypothesis 3: Green bonds contribute to reducing carbon emissions by enhancing the effectiveness of green technology innovation.

## 3. Materials and methods

### 3.1. Research methodology

Using three model selection and construction methods, this paper examines the impact of green bond issuance on carbon emissions.

#### 3.1.1. Spatial autocorrelation test

To assess the spatial correlation between green bond issuance and carbon emission reduction, this study utilizes a comprehensive assessment index—the Global Moran Index, to conduct an analysis. The Global Moran Index formula, as displayed in Eq (1), is used to determine the level of spatial autocorrelation.

$$I=\frac{\sum_{i=1}^{n}\sum_{j=1}^{n}\omega_{ij}\left(x_{i}-\bar{x}\right)\left(x_{j}-\bar{x}\right)}{S^{2}\sum_{i=1}^{n}\sum_{j=1}^{n}\omega_{ij}}$$ (1)

where the sample variance $S^{2}=\frac{\sum_{i=1}^{n}{\left(x_{i}-\bar{x}\right)}^{2}}{n}$, $\bar{x}=\frac{x_{i}}{n}$, *n* indicates total number of regions, *x*_*i*_ represents the observation in the ith region, *ω*_*ij*_ is the spatial weighting element, and $\sum_{i=1}^{n}\sum_{j=1}^{n}\omega_{ij}$ is the sum of all spatial weights. If the spatial weight matrix is transformed into a row normalization,$\sum_{i=1}^{n}\sum_{j=1}^{n}\omega_{ij}=n$. The global Moran’s index value usually falls between -1 to 1, where a value greater than 0 signifies positive autocorrelation, indicating high and high neighborhoods as well as low and low neighborhoods; a value less than 0 indicates negative autocorrelation, implying high and low neighborhoods.

#### 3.1.2. Spatial weight matrix

The basis of exploratory spatial data analysis is the spatial weight matrix; Two methods for constructing the spatial weight matrix are used in this paper.

**Fig 1:**
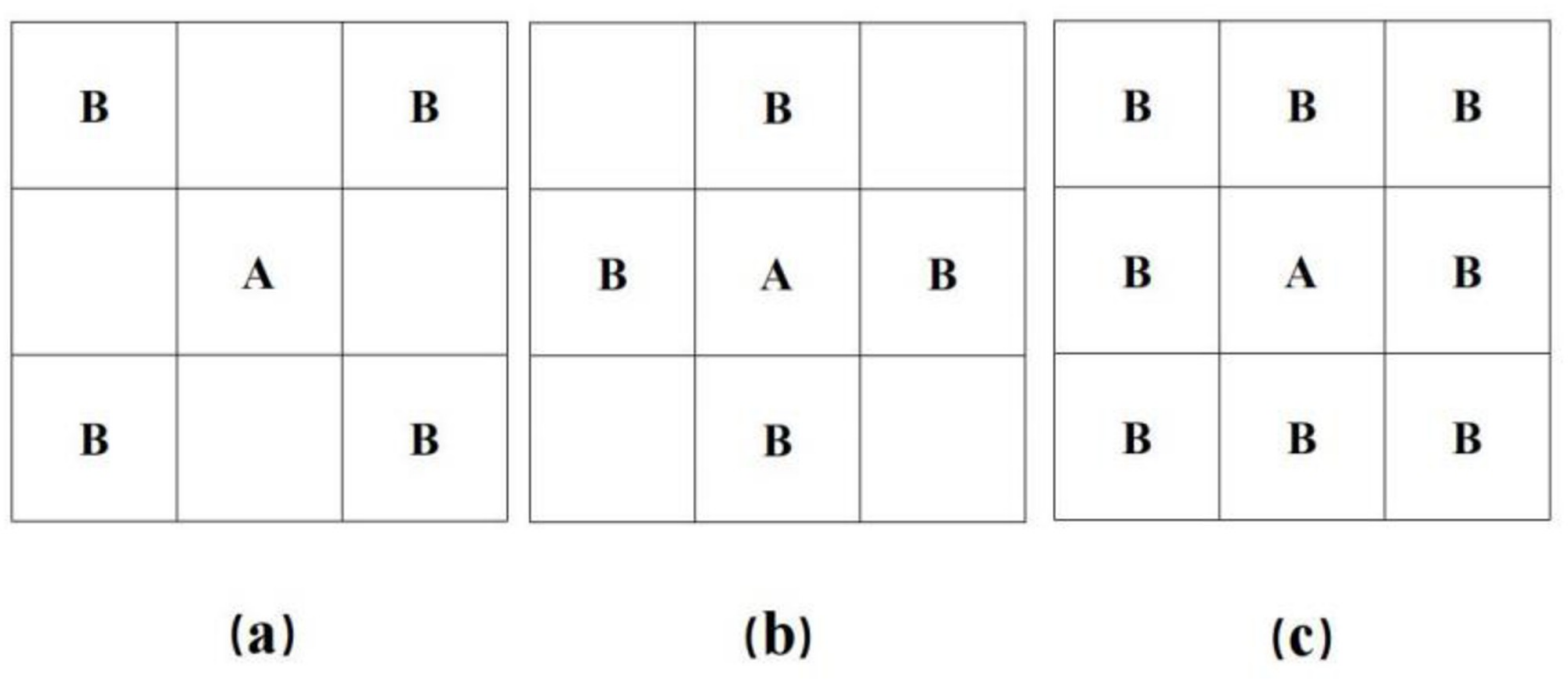
Geographic neighborhood types.

Geographic adjacency matrix: The 0–1 weight matrix, also referred to as a geographic adjacency matrix, effectively describes the degree of association between items. Spatial adjacencies are classified into three types based on common vertices and common boundaries. These adjacencies are displayed in Fig 1 with labeling denoting Bishop, Rock, and Queen adjacencies (a), (b), and (c), respectively.

In this paper, Queen adjacency is chosen and used to construct the following geographic adjacency matrix *W*_1_ to reflect the adjacency between provinces, where the assignment rule for *W*_1_ is as follows:

$$w_{ij}=\left\{\begin{array}{ll} 1 & \text{i},\text{j}\;\text{are}\;\text{adjacent}\\ 0 & \text{i},\text{j}\;\text{are}\;\text{not}\;\text{adjacent}/\text{i}=\text{j} \end{array}\right.$$ (2)

Geographic distance matrix: In this research, the following geographic distance matrix X is constructed to reflect the distance relationship between provinces.

Where *w*_*ij*_ is the inverse of the geographic distance between provinces i and j. For the purposes of the article’s calculations, the capital of each province is specifically used as the province’s representative for the measure.

#### 3.1.3. Spatial measurement models

Since the spatial econometric model can address issues related to spatial explanatory variable autocorrelation and measurement errors, as well as quantify the impact of such variables, this paper uses this technique to evaluate carbon emission impacts of issuing green bonds and its underlying mechanisms. Eq (3) displays the general model of the standard spatial econometric model.


$$\left\{\begin{array}{l} Y_{it}=\lambda Y_{i,t-1}+\rho W_{ij}Y_{it}+\alpha X_{it}+\beta W_{ij}X_{it}+\rho_{i}+\mu_{i}+\varepsilon_{it}\\ \varepsilon_{it}=\varphi W_{ij}\varepsilon_{t}+\nu_{it} \end{array}\right.$$ (3)

Where *Y*_*it*_ and *Y*_*i*,*t*−1_ denote the explanatory variables along with their corresponding first-order lag terms, *X*_*it*_ represents both explanatory and control variables, and *W*_*it*_ refers to the spatial weight matrix. The coefficients for the spatial lag term of the explanatory variables are denoted by *λ*. The spatial autoregressive coefficient is also represented by *ρ*. The regression coefficients of both explanatory and control variables are indicated by *α*. The effects of the spatial lag terms of the explanatory variables and control variables are expressed by *β*. Lastly, the individual and time-fixed effects are represented by *ρ*_*i*_ and *μ*_*i*_ respectively. The random error term is denoted by *ε*_*it*_.

The model is specified as follows:

- A static spatial panel model when *λ* = 0, Otherwise dynamic panel model.
- A spatial Durbin model when *φ* = 0, *α β* ≠ 0;
- Spatial autoregressive model or spatial lag model when *φ* = 0, *β* = 0, *α* ≠ 0;
- Spatial error model when *λ* = *θ* = *α* = 0.

### 3.2. Variable selection

#### 3.2.1. Explained variables

This study examines the impact and mechanism of green bonds on carbon emissions, with the explanatory variable being the issuance of green bonds, referred to as *Greenbonds*. In this study, we retrieved the issuance subject, date, and size of all green bonds issued in China from 2016 to 2021 from the Cathay Pacific database (CSMAR). Following this, we calibrated the data with the applicable provisions of the "Catalogue of Projects Supported by Green Bonds (2021 Edition)". We utilized the website "Tianyancha" to match the registered address of the bond issuers with their respective provinces. We then aggregated and organized the number of green bond issues. in each province throughout the year. Finally, after adding 1 to the result, we performed a natural logarithm transformation.

#### 3.2.2. Explanatory variables

According to the research, the explanatory variable is *Ci*, the carbon emission intensity. *Ci* is calculated as total carbon emissions divided by a region’s actual GDP. It is important to note that technical term abbreviations will be explained upon their first usage. For total carbon emissions in the region, We used the estimation methodology outlined in the 2006 IPCC National GHG Inventory Guidelines. Concurrently, we employed the actual regional gross product derived from each region’s statistical yearbook. The specific formula is as in Eq (4):

$$Ci=\frac{C}{GRP}\times 10^{-4}=\frac{\sum_{j=1}^{8}C_{j}}{GRP}\times 10^{-4}=\frac{\frac{44}{12}\sum_{j=1}^{8}E_{j}\times CF_{j}\times COF_{j}\times EF_{j}}{GRP}\times 10^{-4}$$ (4)

Where *C* represents provincial emissions resulting from combustion of fossil energy; *GRP* represents gross regional product; *j* represents the eight fossil energy types, namely coal, natural gas, coke, fuel oil, gasoline, kerosene, diesel oil, and crude oil; *E* represents the consumption of fossil energy converted to standard coal; *CF* represents the average calorific value of various fuel types; *COF* represents the carbon oxidation factor; and *EF* represents the amount of carbon per unit of calorific value. Since the molecular weights of carbon and carbon dioxide are 12 and 44, respectively, 44/12 must be multiplied to the entire equation to represent the amount of carbon dioxide produced by burning more carbon in oxygen.

The regulations for the average heat output, carbon oxidation factor, and carbon content per unit of calorific value of eight fossil energy fuels are specified in Table 1 below.

**Table 1:** Summary of fossil fuel properties.

Energy	Fuel calorific value	Carbon oxidation rate (%)	Carbon content per unit calorific value (tC/GJ)
Coal	20908 KJ/kg	94	0.02637
Natural gas	32238~38931 KJ/m3	99	0.01532
Coke	28435 KJ/kg	93	0.02950
Fuel oil	41816 KJ/kg	98	0.02110
Gasoline	43070 KJ/kg	98	0.01890
Kerosene	43070 KJ/kg	98	0.01960
Diesel oil	42652 KJ/kg	98	0.02020
Crude oil	41816 KJ/kg	98	0.02008

**Fig 2:**
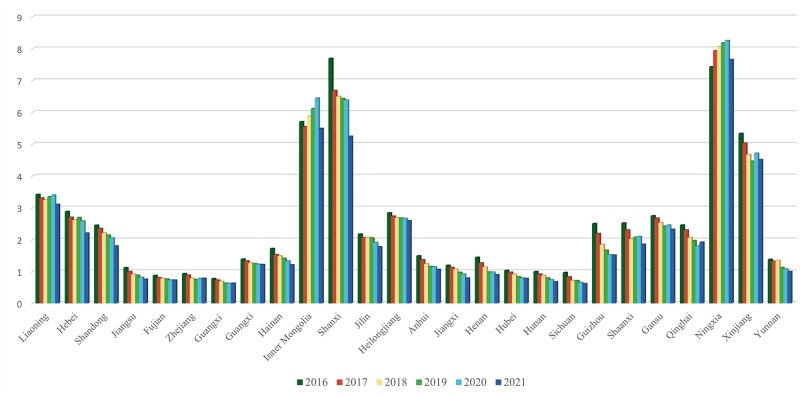
Carbon emission intensity of 26 provinces in China, 2016–2021.

The carbon emission intensity values, as measured by the model and calculation method described above, are presented in Fig 2.

#### 3.2.3. Control variables

Regarding the existing literature, this paper sets the following control variables: (1) Economic development level (GDP): Carbon emission intensity is directly affected by the level of economic development, which is measured by the natural logarithmic value of per capita GDP; (2) Industrial structure (SECOND, THIRD): the main task of industrial structure change is to realize carbon peak carbon neutral, and ultimately to reduce the intensity of carbon emissions, which is measured by the proportion of value added of secondary and tertiary industries to regional GDP; (3) Scale of urban development (UPD, ED): the scale of urban development affects the carbon emission intensity through both the size of population and the strength of economic development. Measured by population density (the natural logarithm of urban population density) and economic density (the natural logarithm of the ratio of gross regional product to the total regional population at the end of the year), respectively; (4) Scale of investment (INVEST): the scale of investment will indirectly affect carbon emission intensity by influencing the survival and development of enterprises, as measured by the growth rate of investment in fixed assets (excluding agricultural households); (5) Environmental regulation (ER): environmental regulation can also significantly affect the intensity of urban carbon emissions, measured by the logarithm of the amount of investment in pollution control; (6) Foreign investment (FOREIGN): foreign investment policies will promote economic development by generating different attractions, and ultimately affect the intensity of carbon emissions, expressed as the logarithm of the total amount of investment in foreign-invested enterprises.

#### 3.2.4. Intermediary variables

Based on the theoretical analysis, it was determined that issuing green bonds can lead to a reduction in carbon emissions by altering energy consumption patterns and facilitating the adoption of green technological innovations among large-scale industrial enterprises. As a result, this paper has chosen energy consumption patterns and green technological innovations as intermediary variables. The following section provides specific details on these variables.

Energy consumption structure: Based on the above theoretical analysis, the ECS variable measures the structure of energy consumption by the ratio of the consumption of coal to the total consumption of energy.

Green technology innovation efficiency: Three main types of measurement methods for assessing the efficiency of green technology innovation emerge from the reading and organizing of the available literature: direct measurement via regional green patents data aggregation; measurement through construct of an indicator system combined with the entropy value method; and measurement with the DEA method. Upon comprehensive analysis of the three analyses, recognize that the development of the initial indicator system can suffer from the biases of users, making it impossible to examine and derive the genuine innovation efficiency of the area objectively and accurately. Furthermore, the second analysis is hindered by the absence of corresponding data on green patents of industrial enterprises on a large scale, thereby constraining the ability to determine corresponding values. For the issue of non-anticipated outputs in the third method, utilizing the conventional DEA model is challenging and cannot be adequately addressed. Therefore, this study employs the non-radial, non-angle, non-expected output SBM-DEA model developed by Tone (2001) to assessment of the effectiveness of green technology innovation in major industrial companies in each province, Expressed in *GTI*. The measurement process is outlined as follows:

Assume that each province is a Production Decision Unit, encompassing three input variables (*X*), desired outputs (*Y*^*g*^), and non-desired outputs (*Y*^*b*^), the matrices of *X*, *Y*^*g*^, *Y*^*b*^ are outlined as follows: *X* = (*x*_1_, *x*_2_, ⋯, *x*_*n*_) ∈ *R*^*m*×*n*^, $Y^{g}=\left(y_{1}^{g},y_{2}^{g},\cdots ,y_{n}^{g}\right)\in R^{s_{1}\times n}$, $Y^{b}=\left(y_{1}^{b},y_{2}^{b},\cdots ,y_{n}^{b}\right)\in R^{s_{2}\times n}$, where *X* > 0, *Y*^*g*^ ≥ 0, *Y*^*b*^>0 respectively. The set of production possibilities *P* can be defined as:

$$P=\left\{\left(x,y^{g},y^{b}\right)\left|x\geq \lambda X,y^{g}\leq \lambda Y^{g},y^{b}\geq \lambda Y^{b},\lambda \geq 0\right.\right\}$$ (5)

In Eq (5), *λ* denotes the weight vector of cross-sectional observations, and the corresponding SBM model for a specific production decision unit *DMU*_0_ is formulated as follows:

$$\begin{array}{l} P^{*}=\frac{1-\frac{1}{m}\sum_{r=1}^{m}\frac{s_{i}^{-}}{x_{i_{0}}}}{1+\frac{1}{s_{1}+s_{2}}\left(\sum_{r=1}^{s_{1}}\frac{s_{r}^{g}}{y_{r_{0}}^{g}}+\sum_{r=1}^{s_{2}}\frac{s_{r}^{b}}{y_{r_{0}}^{b}}\right)}\\ s.t.\left\{\begin{array}{l} X_{0}=X_{\lambda }+s^{-}\\ y_{0}^{g}=Y^{g}\lambda -s^{g}\\ y_{0}^{b}=Y^{b}\lambda +s^{b}\\ \lambda ,s^{-},s^{g},s^{b}\geq 0 \end{array}\right. \end{array}$$ (6)

In Eq (6), P* fulfills 0 ≤ P* ≤ 1, which states that the efficiency of green technological innovation in large-scale industrial enterprises is a strictly decreasing function with respect to the three slack variables $s^{-}$ *S*^*g*^, *S*^*b*^. Here, $s^{-}$ represents surplus inputs, *S*^*g*^ expresses insufficient desired outputs, and *S*^*b*^ indicates excess non-desired outputs.

The specific content framework for the above variables *X*, *Y*^*g*^ and *Y*^*b*^ is shown in Table 2 below.

**Table 2:** Green technology innovation index system.

Type of indicator	Variable	Meaning
Green Technology Innovation Input Indicators	Labor Force	Full-time equivalents of research and experimental development personnel (person-years)
	Capital	Stock of internal expenditure on research and experimental development (million)
		Funding for new product development (million)
	Energy	Total energy consumption (tons of standard coal)
Green Technology Innovation Output Indicators	Expected output	Income from new product sales (million)
		Authorized invention patents (pieces)
	Non-expected output	Comprehensive urban environmental pollution index

Regarding the calculation of the urban environmental pollution index, the representative data of a province is taken as the capital city, and it is combined with the corresponding data on pollutant emissions from the China Environmental Statistical Yearbook. The ambient air pollutant concentration standards for the basic items (as presented in Table 3) are used to calculate the composite pollution index. Additionally, specific data for all other variables are directly obtained from the relevant statistical yearbooks.

**Table 3:** Ambient air pollutant concentration standards (in China).

Pollutant Item	Averaging time	Concentration Limit		Unit
		Level 1	Level 2	
Sulfur Dioxide	Annual average	20	60	*μg*/*m*^3^
	24-hour average	50	150	
	1 hour average	150	500	
Nitrogen Dioxide	Annual average	40	40	
	24-hour average	80	80	
	1 hour average	200	200	
Carbon Monoxide	24-hour average	4	4	*mg*/*m*^3^
	1 hour average	10	10	
Ozone	Daily maximum 8-hour average	100	160	*μg*/*m*^3^
	1 hour average	160	200	
Particulate matter (particle size ≤10μm)	Annual average	40	70	
	24-hour average	50	150	
Particulate matter (particle size ≤2.5μm)	Annual average	15	35	
	24-hour average	35	75	

### 3.3. Data sources and descriptive statistics

In this paper, 26 provinces in China are used as the basic research samples from 2016–2021, and the sources mainly include (1) The data concerning green bonds is primarily sourced from the Cathay Pacific Database (CSMAR). (2) Data on carbon emissions is mainly obtained from the China Energy Statistics Yearbook, China Statistics Yearbook, and the Carbon Emissions Trading Network. (3) Control variable data is primarily sourced from the China Statistics Yearbook and China Environmental Statistics Yearbook. (4) Mediator variable data is obtained from the China Science and Technology Statistics Yearbook, China Energy Statistics Yearbook, China Industrial Statistics Yearbook, and China Environmental Statistics Yearbook.

Based on the unique policy making and economic development characteristics of municipalities, this paper excludes four municipalities from the sample of 31 provinces. Additionally, Tibet is excluded due to the absence of multiple sets of data, which makes it impossible to precisely measure the region’s development status, predict developmental trends, and renders the data unrepresentative. In summary, we have selected the remaining 26 provinces in China as the research sample to ensure the universality of the findings. By utilizing the calculation methods and fundamental data, descriptive statistics for the key variables are presented in Table 4.

**Table 4:** Descriptive statistics for all variables.

Variable	Sample size	Mean	Standard Deviation	Min	Max
*Greenbonds*	156	2.89	2.13	0	6.51
*Ci*	156	2.28	1.91	0.61	8.25
*GDP*	156	10.95	0.32	10.23	11.83
*SECOND*	156	0.39	0.06	0.19	0.50
*THIRD*	156	0.50	0.04	0.40	0.62
*UPD*	156	7.95	0.35	7.20	80.62
*ED*	156	10.93	0.32	10.26	11.83
*INVEST*	156	0.06	0.10	-0.63	0.21
*ER*	156	5.48	1.10	1.48	7.48
*FOREIGN*	156	7.06	1.37	4.32	10.72
*ECS*	156	0.71	0.33	0.24	1.72
*GTI*	156	0.61	0.23	0.26	1

## 4. Results

### 4.1. Model checking

According to the specific research methodology outlined in this paper, the model testing encompasses two primary aspects: the spatial autocorrelation test and the spatial econometric model test.

#### 4.1.1. Spatial autocorrelation test

**Fig 3:**
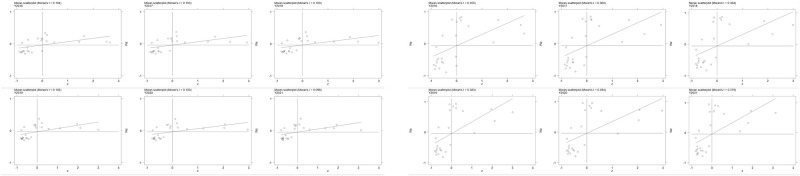
Sub-area Moran’s Index under Geographic Distance (left) / Neighborhood (right) Matrix.

The Moran index represents the correlation coefficient between observed values and their spatial lags, which are displayed in a scatter plot known as the "Moran scatter plot". Specific test results are shown in Fig 3. The results shown in Table 5 are obtained by calculating the global Moran’s index as specified.

**Table 5:** Results of spatial autocorrelation tests.

Year	*W*_1_		*W*_2_	
	*Greenbonds*	*Ci*	*Greenbonds*	*Ci*
2016	0.243** (2.196)	0.355*** (3.173)	0.033*** (2.134)	0.104*** (4.378)
2017	0.033 (0.550)	0.360*** (3.237)	0.041*** (2.323)	0.103*** (4.371)
2018	0.434*** (3.608)	0.364*** (3.291)	0.095*** (3.885)	0.100*** (4.296)
2019	0.376*** (3.168)	0.383*** (3.448)	0.084*** (3.579)	0.102*** (4.356)
2020	0.383*** (3.192)	0.384*** (3.452)	0.116*** (4.451)	0.100*** (4.280)
2021	0.398*** (3.402)	0.379*** (3.436)	0.110*** (4.414)	0.098*** (4.263)

Standard errors are shown in parentheses;

*, **, and *** denote p < 0.1, p < 0.05, and p < 0.01, respectively (Same table as below)

The analysis presented in Table 5 indicates that the global Moran indexes for both green bonds and carbon emission reduction are greater than 0, with most reaching the significance level of 1%. The results show a clear link between green bond issuance and reduced carbon emissions in Chinese provinces. At the same time, with few exceptions, the overall global Moran’s index of green bonds is higher under the geographic neighbor matrix compared to the geographic distance matrix. This suggests that geographic factors have a strengthened impact on the positive spatial dependence of green bonds.

#### 4.1.2. Spatial econometric model testing

The model underwent LM, Hausman, LR, and Wald tests, as reflected in Table 6.

**Table 6:** Results of spatial measurement model tests.

Model Checking	*W*_1_		*W*_2_	
	Value	P-Value	Value	P-Value
*LM-lag*	13.444***	0.000	1.819	0.177
*Robust LM-lag*	11.379***	0.000	1.637***	0.000
*LM-error*	4.630**	0.031	6.806***	0.009
*Robust LM-error*	4.905**	0.027	6.624***	0.001
*Hausman-test*	110.45***	0.0000	167.46***	0.0000
*LR-test-ind*	7.36	0.8237	23.14	0.3671
*LR-test-time*	324.83***	0.0000	315.26***	0.0000
*LR-test (SAR)*	29.33***	0.0006	41.47***	0.0000
*LR-test (SEM)*	42.12***	0.0000	44.69***	0.0000
*Wald-test (SAR)*	15.50*	0.0502	39.54***	0.0000
*Wald-test (SEM)*	31.72***	0.0002	56.94***	0.0000

On the basis of the findings in Table 6, this study selects the fixed effects model as it is indicated by the significant Hausman test statistic at the 1% level under both types of matrices. The LR-test results also suggest that the time fixed effect is chosen. Finally, the LR-test and Wald-test results indicate that the statistics for both tests in SAR and SEM are significant at the 5% level. This suggests that the SDM model cannot be converted into an SAR or SEM model.

From the above analysis, the current study selects a spatial Durbin model (SDM) with fixed time effects to examine the impact of green bond issuance on reducing carbon emissions. Eq (7) demonstrates the specific model used.


$$\begin{array}{l} Ci_{it}=\lambda Ci_{i,t-1}+\omega WCi_{it}+\alpha_{1}Greenbonds_{it}+\sum_{n=1}^{8}a_{n}control_{it}^{n}+\beta_{1}WGreenbonds_{it}\\ +\sum_{n=1}^{8}b_{n}Wcontrol_{it}^{n}+\mu_{i}+\upsilon_{it} \end{array}$$ (7)

Where *Ci* represents carbon emission intensity, *Greenbonds* indicates the amount of green bonds that have been issued, and control denotes the control variables, which contain eight variables, *GDP*, *SECOND*, *THIRD*, *UPD*, *ED*, *INVEST*, *ER* and *FOREIGN*, respectively. *λ*, *ω*, *α*_*n*_, *β*_*n*_ represents the parameter under estimation and *W* is the spatial matrix. *μ*_*i*_, *υ*_*it*_ denote time fixed effects and random error terms, respectively It is important to highlight that if *λ* equals 0, Eq (7) represents a static spatial Durbin model, and if *λ* does not equal 0, Eq (7) represents a dynamic spatial Durbin model.

### 4.2. Analysis of model results

#### 4.2.1. Analysis of spatial effects

To examine the direct impact and spatial spillover consequences of green bond issuance on carbon emissions, the SDM model is utilized in this study to test Hypothesis 1. Analyzing the regression coefficients from the experiment, we find that, regarding the issues examined in this study, whether through analyzing the geographic neighbor matrix or the geographic distance matrix, the coefficients for green bond issuance are significantly negative in both the static and dynamic models. The issuance of green bonds has the potential to improve society’s focus on green environmental protection, increase environmental protection efforts, reduce air pollutant emissions, and strengthen air pollution control measures. Rectifying measures should strengthen the control of air pollutant emissions while improving the quality standards of enterprises in their daily operations. The combination of these approaches will reduce carbon emissions and verify hypothesis 1.

In the dynamic spatial Durbin model with explanatory variables such as the geographic proximity and distance matrices, the coefficients of the time lag terms for carbon emissions are significantly greater than 0. The current carbon emission intensity positively promotes the strengthening of the intensity of carbon emissions in the following period. This finding suggests that reducing carbon emissions is not a short-term task in the process of development but is a long-term concern. The coefficient of the relevant spatial lag term is not significant in all cases except for the geographic adjacency matrix under the static model. This implies that carbon emissions of neighboring regions only have a driving effect on each other’s emissions in the same period, but the effect remains unchanged by the distance between them. Simultaneously, the current period’s carbon emission intensity does not affect the carbon emission intensity of neighboring regions in later periods. As such, each region must establish a development and management system that is appropriate for its unique circumstances, in conjunction with the central government’s core policies, and devise effective policy measures that align with local conditions.

#### 4.2.2. Analysis of intermediation effects

After the theoretical analysis, the mediating effect between green bond issuance and carbon emissions is illustrated. To test hypotheses 2 and 3, we utilize Wen et al. (2022) [54] simple mediation effect model in conjunction with the model (7).

In this paper, the method corresponding to the above model is applied for testing, and the results of the test are presented in Table 7 below.

**Table 7:** Results of the regression analysis of the mediation effect.

Panel A	*W*_1_/*W*_2_			Panel B	*W*_1_/*W*_2_		
	*Ci*	*ECS*	*Ci*		*Ci*	*ECS*	*Ci*
*Greenbonds*	−0.452*** (−7.27)	−0.028** (−2.25)	−0.368*** (−7.26)	*Greenbonds*	−0.452*** (−7.27)	0.037*** (4.51)	−0.390*** (−6.03)
*ECS*			3.015*** (9.34)	*GTI*			−1.694*** (−2.81)
*_cons*	3.589*** (16.09)	0.788*** (17.68)	1.213*** (3.90)	*_cons*	3.589*** (16.09)	0.504*** (17.31)	4.443*** (11.87)
control variable	*Yes*	*Yes*	*Yes*	control variable	*Yes*	*Yes*	*Yes*
sample size	*156*	*156*	*156*	sample size	*156*	*156*	*156*

In the above analysis, the results for examining the structure of energy consumption and the efficiency of green technology innovation are presented in Panels A and B, respectively. These results are combined for analysis and discussion due to the shared effect of either the geographic adjacency matrix or the geographic distance matrix in mediating effect analysis.

As evident in Panel A, the analysis of the model reveals significant results for both green bonds and energy consumption structure at the 5% level. The coefficients for green bonds are significantly negative in the outcome, signifying a crucial role of green bond issuance in curbing carbon emissions. At the same time, it is evident that issuing green bonds can affect energy consumption patterns. The structure of energy consumption, which denotes the proportion of coal consumption in total energy consumption, can be altered with the issuance of green bonds. This can result in reduced coal consumption, thus positively impacting carbon emissions. After including the energy consumption structure variable, the coefficient of green bonds changed from -0.452 to -0.368, indicating a suppressed effect and a reduced absolute value. The impact of green bonds on carbon emissions can be mitigated by the structure of energy consumption. To conclude, the analysis confirms that issuing Green Bonds can help change the structure of energy consumption and lead to reduced carbon emissions.

According to Panel B, both green bonds and green technology innovation efficiency are statistically significant at the 1% level. The coefficient of green bonds is significantly negative according to the results, which is in line with Panel A’s analysis. The conclusion infers that the issuance of green bonds improves the efficiency of green technology innovation. In addition, carbon emissions are significantly reduced through the effectiveness of green technology innovation. After incorporating the energy consumption structure, the coefficient of green bonds shifted from -0.452 to -0.390, resulting in a reduction of its absolute value. This indicates that green technology innovation efficiency can also mitigate the impact of green bonds on carbon emissions. Overall, the analysis suggests that issuing green bonds can improve the effectiveness of innovative green technologies, thereby reducing carbon emissions. The verification of hypothesis 2 and 3 is a successful outcome of the above analysis.

#### 4.2.3. Robustness check

To ensure objectivity in our analysis, this paper concentrates on testing the robustness of the spatial Durbin model. We utilize two methods, panel-corrected standard error estimation and lagged multi-period regression, to conduct the assessments.

Panel-corrected standard error estimates: The test outcomes are presented in S1 Appendix. According to the test results, the P-value of heteroskedasticity and autocorrelation test is 0, which indicates the existence of heteroskedasticity and autocorrelation in the sample data. The regression coefficient of green bonds was found to be still significantly negative in the results after estimation using PCSE, which is consistent with the results of spatial effect analysis, indicating that the model has reliable stability.

Lagged multi-period regression: The previous model Eq (7) underwent the lagged multi-period regression process, and the test outcomes are presented in S2 Appendix.

As shown by the test results, the regression coefficients for carbon emissions in the lagged period are significantly positive at the 1% level for both the geographic adjacency and distance matrices. Over the next three periods, these coefficients continued to show significant positivity at the 1% level. Thus, this implies that the magnitude of carbon emissions in the previous period had a notable influence on the following period’s intensity. In the regression results spanning from one lagged period to three periods, in most cases, the regression coefficients of green bonds are not significant at the 10% significance level. However, without lagging, the regression coefficients of green bonds are significant at the 10% significance level. This indicates to a certain extent that the model is smooth.

#### 4.2.4. Heterogeneity analysis

Given the impact of economic development, the 26 sampled provinces will be categorized into economically developed and underdeveloped regions according to their per capita GDP, in compliance with the standard set by the China Statistical Yearbook. Each group was regressed using Eq (6), as shown in Table 8 below.

**Table 8:** Results of the heterogeneity test based on the degree of economic development.

Variable	Economically developed Regions		Less economically developed regions	
	*W*_1_	*W*_2_	*W*_1_	*W*_2_
*Greenbonds*	−0.251*** (−3.86)	−0.183** (−2.20)	−0.288** (−2.39)	−0.309*** (−2.82)
*W×Greenbonds*	−0.052 (−1.34)	−0.687*** (−2.69)	−0.182* (−1.80)	−1.073*** (−2.99)
*Year*	*Yes*	*Yes*	*Yes*	*Yes*
*N*	42	42	114	114
*R* ^2^	0.1968	0.1901	0.3911	0.4440

From the findings in Table 8, it is evident that green bonds have a significant negative correlation with carbon emissions at the 5% level for both economically developed and underdeveloped regions, given the two types of matrices. However, in the economically underdeveloped regions, the absolute value of the coefficient of the impact of green bonds and the coefficient of the spatial interaction term under the conditions of the two types of matrices is greater than that of the economically developed regions. This indicates that green bond issuance has a stronger inhibitory effect on carbon emissions in economically underdeveloped regions compared to economically developed regions. In economically developed regions, the presence of multiple emission mitigation mechanisms and the influence of numerous factors may reduce the impact of green bonds on carbon emissions. Conversely, in economically underdeveloped regions with fewer pathways, the role played by regional green bonds on emissions reduction is more pronounced. Furthermore, when the coefficients of the spatial interaction terms of green bonds in the two regions are combined, it is evident that the spatial regression coefficients of both regions are significantly negative at the 1% level when based on the geographic distance matrix. In addition, the coefficients of the economically developed regions are not significant, while those of the economically underdeveloped regions are significantly negative at the 10% level when based on the geographic neighboring matrix. Based on the geographic distance matrix cell values, which are inversely proportional to the distance between regions, our analysis shows that neighborhoods that issue green bonds substantially reduce local carbon emissions. This effect changes inversely with geographic distance and is particularly noted in provinces with lower economic development. Interestingly, this effect was not observed in economically developed regions.

## 5. Discussion

The issuance of green bonds can impact carbon emission intensity in two ways. Firstly, green bonds can promote the conversion of consumption structure and convert environmental pollution into financing costs by stimulating enterprises’ green awareness and promoting equipment technology upgrading (Liu and Ren, 2019). At the same time, capital flow is also guided and channeled towards relevant green enterprises through green bonds to promote the construction of green and low-carbon industries. This will improve the structure of energy consumption and reduce carbon emission intensity. On the other hand, green bonds can promote the efficiency of green technological innovation. However, the technology itself has some disadvantages, such as high risk, high investment, and long cycles, which can lead to credit constraints. Additionally, environmental regulations can cause a crowding-out effect. However, green bonds can alleviate financing constraints and optimize the internal debt composition of enterprises, promoting technological innovation efficiency and reducing carbon emissions. As the economy rapidly develops, green bonds are increasingly playing a role in global green development. The empirical model’s test results indicate that a 1% increase in green bond issuance leads to a reduction of 0.306% and 0.331% in carbon emission intensity based on the geographic proximity matrix and geographic distance matrix, respectively. This suggests that green bond issuance can effectively reduce carbon emission intensity.

The impact of green bond issuance on carbon emission intensity varies across different economic development regions. From a spatial perspective, it can be analyzed that the issuance of green bonds under different levels of economic development does not have the same impact on carbon emissions in neighboring provinces. Economically developed areas tend to have more technological advancements and resources, resulting in a greater number of options for reducing carbon emissions. This includes a wider range of green bond alternatives, which can help to reduce the impact of green bonds on carbon emissions in the local area. As a result, the inhibitory effect of green bond issuance on carbon emissions is stronger in economically underdeveloped areas compared to economically developed areas. Additionally, economic development can impact the issuance of green bonds. The spillover effect of green bond issuance in the region can have varying degrees of influence on surrounding areas. Learning to efficiently use new environmentally friendly tools to improve the environment while promoting rapid economic development through natural symbiosis, in order to achieve efficient ecological protection.

At the same time, it is necessary to recognize three limitations of this work. The first is that in the selected interval, the new crown epidemic that began in early 2020 had a significant impact on economic development, leaving the representativeness of the data for 2020 and 2021 to be demonstrated. Second, the selected interval is six years in total, which is a short interval based on the availability of existing data. Third, there is the problem of selecting intermediate variables. The selected energy consumption structure and green technology innovation efficiency can be analyzed by theory and empirical evidence, but there may be other transmission channels. Therefore, based on the above limitations, in the future research work, the sample data of the new crown epidemic interval will be analyzed and compared separately to eliminate the influence of the epidemic period to improve the accuracy of the conclusion. For the length of intervals, with the gradual release of statistical yearbooks and related information, the study period will be gradually extended to enhance the persuasiveness of the article. For the analysis of transmission path research, in the follow-up work, all possible ways of influence will be considered as comprehensively as possible, in order to analyze the transmission path between the two in a more complete way.

## 6. Conclusions and police recommendations

This study analyzes sample data from 26 Chinese provinces issued between 2016 and 2021, in terms of green bonds and carbon emissions, combined with the spatial Durbin model to empirically analyze and study the relationship between the impact of green bond issuance on carbon emissions, and at the same time, add the mediating effect to analyze the impact of green bonds on the path of carbon emissions, combined with the empirical results of the analysis of the findings: (1) Issuing green bonds negatively correlates with regional carbon emissions under varying spatial weight matrices, suggesting that emitting less carbon is associated with a higher number of issued green bonds. Additionally, empirical analysis reveals that the intensity of carbon emissions in the subsequent period is positively driven by the current period’s carbon emission intensity, and neighboring regions’ carbon emissions in the current period have a driving effect on the region; (2) Green bonds can help reduce carbon emissions by adjusting energy consumption structures or improving the efficiency of green technology innovation; (3) Further analysis shows that, due to the more advanced and rich technology in developed regions, they can produce a greater effect in reducing carbon emissions compared to the impact of economic development alone. This means that green bonds are a more effective alternative to reduce carbon emissions, which also reduces the impact of green bonds on carbon emissions unilaterally in the local area. Therefore, the inhibition effect of carbon emissions from green bond issuance in less developed regions is stronger than in economically developed regions. Additionally, in economically underdeveloped regions, neighboring regions’ issuance of green bonds significantly inhibits carbon emissions in the area, while this effect is not observed in economically developed regions.

Based on the conclusions drawn from the above studies, the following recommendations are targeted respectively: (1) Continue to advance the growth of the green bond market in adherence to the original direction. Avoid tailoring to societal and market shifts, and adeptly employ the green tools of the contemporary era in environmental enhancement, ultimately bolstering ecological protection efficacy. At the same time, we should give consideration to the objective disclosure of information and establish stricter environmental disclosure requirements as part of the internal assessment process for businesses. This will encourage a more standardized market and maximize the effectiveness of green bonds in promoting environmental governance. (2) Various measures can be implemented to enhance the drive towards high-quality economic growth and to facilitate the expeditious advancement of society. From a societal standpoint, tax incentives related to green and low-carbon areas can be adjusted to facilitate financing expansion, promote increased attention and participation in green development, and popularize green development. Additionally, financing methods for enterprises can be improved during development by increasing the proportion of green bonds and leveraging the benefits of sustainable development to enhance competitive advantage. (3) Formulate policies and systems for green reforms that are suitable for the practical and localized development of the region, considering the level of marketization and environmental protection in the current context. One approach is to strengthen penalties for environmental pollution through legislation and other means. Another approach is to deepen financial system reforms to ease financing constraints associated with green technological innovation. Additionally, promoting green investment and financing can facilitate multi-faceted joint efforts towards coexistence of humanity and the environment.

## Supporting information

S1 Dataset (XLSX)

S1 Appendix Panel-corrected standard error estimation test results. (DOCX)

S2 Appendix Model lagged multi-period regression results. (DOCX)

## References

[1] Dasgupta S, Laplante B, Mamingi N. Pollution and capital markets in developing countries. Journal of Environmental Economics and Management. 2001; 42(3):310–335. doi: 10.2139/ssrn.124948

[2] Shahbaz M, Solarin SA, Mahmood H, Arouri M. Does financial development reduce CO_2_ emissions in Malaysian economy? A time series analysis. Economic Modelling. 2013; 35:145–152. doi: 10.1016/j.econmod.2013.06.037

[3] Shao HH, Liu YB. The nonlinear relationship between financial development and carbon emission—Based on panel smooth transition regression model. Soft Science. 2017; 31(05):80–84. doi: 10.13956/j.ss.1001-8409.2017.05.18

[4] Shahbaz M, Nasir MA, Roubaud D. Environmental degradation in France: The effects of FDI, financial development, and energy innovations. MPRA Paper. 2018; 74:843–857. doi: 10.1016/j.eneco.2018.07.020

[5] Muzzammil H, Wang W, Wang YW. Natural resources, consumer prices and financial development in China: Measures to control carbon emissions and ecological footprints. Resources Policy. 2022; 78:102880. doi: 10.1016/J.resourpol.2022.102880

[6] Salahuddin M, Gow J, Ozturk I. Is the long-run relationship between economic growth, electricity consumption, carbon dioxide emissions and financial development in Gulf Cooperation Council Countries robust? Renewable and Sustainable Energy Reviews. 2015; 51:317–326. doi: 10.1016/j.rser.2015.06.005

[7] Dogan E, Seker F. The influence of real output, renewable and non-renewable energy, trade and financial development on carbon emissions in the top renewable energy countries. Renewable and Sustainable Energy Reviews. 2016; 60:1074–1085. doi: 10.1016/j.rser.2016.02.006

[8] Yan CL, Li T, Lan W. Financial development, innovation and carbon emission. Journal of Financial Research. 2016; (01):14–30.

[9] Huang YM, Xue L, Khan ZS. What abates carbon emissions in China: Examining the impact of renewable energy and green investment. Sustainable Development. 2021; 29(5):823–834. doi: 10.1002/SD.2177

[10] Jiang HL, Wang WD, Wang L, Wu JH. The effects of the carbon emission reduction of China’s green finance—An analysis based on green credit and green venture investment. Finance Forum. 2020; 25(11):39–48+80. doi: 10.16529/j.cnki.11-4613/f.2020.11.006

[11] Arshian S, Najia S, Dong KY, Khan S. Nexus between green technology innovation, green financing, and CO_2_ emissions in the G7 countries: The moderating role of social globalisation. Sustainable Development. 2022; 30(6):1934–1946. doi: 10.1002/sd.2360

[12] Wan YY, Sheng N. Clarifying the relationship among green investment, clean energy consumption, carbon emissions, and economic growth: a provincial panel analysis of China. Environmental Science and Pollution Research International. 2021; 29(6):9038–9052. doi: 10.1007/s11356-021-16170-w 34498185

[13] Cao TQ, Zhang CY, Yang X. Green effect and influence mechanism of green credit policy—Based on the evidences of green patent data of Chinese listed companies. Finance Forum. 2021; 26(05):7–17. doi: 10.16529/j.cnki.11-4613/f.2021.05.003

[14] Sun HP, Chen TT, Wang CN. Spatial impact of digital finance on carbon productivity. Geoscience Frontiers. 2023; 101674. doi: 10.1016/j.gsf.2023.101674

[15] Guo W, Sun T. Effect of population structure change on carbon emission in China: Based on perspectives of urbanization and residents ‘consumption. Journal of Applied Statistics and Management. 2017; 36(02):295–312. doi: 10.13860/j.cnki.sltj.20160722-002

[16] Anser KM, Alharthi M, Aziz B, Wasim S. Impact of urbanization, economic growth, and population size on residential carbon emissions in the SAARC countries. Clean Technologies and Environmental Policy. 2020; 22(4):1–14. doi: 10.1007/s10098-020-01833-y

[17] Zhang CL, Zhang F. The impact of ecological protection and industrial structure upgrading on carbon emissions-An empirical study based on the data of the Yangtze River Economic Belt. Statistics and Decision. 2022; 38(03):77–80. doi: 10.13546/j.cnki.tjyjc.2022.03.014

[18] Qu XE, Luo HY. Impact of China’s OFDI on carbon emissions and its transmission mechanism: an empirical analysis based on multiple mediation effect model. China Population, Resources and Environment. 2021; 31(07):1–14.

[19] Zhang S, Liu X, Bae J. Does trade openness affect CO_2_ emissions: evidence from ten newly industrialized countries? Environmental Science and Pollution Research International. 2017; 24(21):17616–17625. doi: 10.1007/s11356-017-9392-8 28597097

[20] Zhang TF, Yang J, Sheng PF. The impacts and channels of urbanization on carbon dioxide emissions in China. China Population. Resources and Environment. 2016; 26(02):47–57.

[21] Qi X, Guo XN. A comparative study of the impact of urbanization on carbon emissions in emerging economies. Ecological Economics. 2022; 38(03):101–108.

[22] Li YY, Huang LJ. Analysis of the spatial spillover effect of fiscal environmental protection expenditure on carbon emission reduction. Statistics and Decision. 2022; 38(15):154–158. doi: 10.13546/j.cnki.tjyjc.2022.15.029

[23] Song X, Jia JS, Ju M. Provincial contributions analysis of the slowdown in the growth of China’s industrial CO_2_ emissions in the "New Normal". Environmental Science, Economics. 2021; doi: 10.15244/pjoes/129689

[24] Liu ST, Jia JS, Huang HZ, Zhong YX, Zhou YM. China’s CO_2_ Emissions: A thorough analysis of spatiotemporal characteristics and sustainable policy from the agricultural land-use perspective during 1995–2020. Environmental Science, Economics, Agricultural and Food Sciences. 2023; 12(6): doi: 10.3390/land12061220

[25] Jia JS et al. China’s CO_2_ emissions: An innovative framework for analyzing carbon reduction in sustainable tourism under the guidance of the United Nations’ sustainable development goals. Journal of Cleaner Production. 2023; 430:139752. doi: 10.1016/j.jclepro.2023.139752

[26] Britta H, Dirk S. Are green bonds priced differently from conventional bonds? Journal of Asset Management. 2018; 19(6):371–383. doi: 10.1057/s41260-018-0088-5

[27] Zerbib DO. The effect of pro-environmental preferences on bond prices: Evidence from green bonds. Journal of Banking and Finance. 2018; 98:39–60. doi: 10.1016/j.jbankfin.2018.10.012

[28] Wang JZ, Chen X, Li XX, Yu J, Zhong R. The market reaction to green bond issuance: Evidence from China. Pacific-Basin Finance Journal. 2020; 60(Prepublish):101294–101294. doi: 10.1016/j.pacfin.2020.101294

[29] Kapraun J, Scheins C. (In)-Credibly Green: Which Bonds Trade at a Green Bond Premium? Paris December 2019 Finance Meeting. 2019;.

[30] Hyun S, Park D, Tian S. The price of going green: the role of greenness in green bond markets. Accounting & Finance. 2020; 60(1):73–95. doi: 10.1111/acfi.12515

[31] Ehlers T, Packer F. Green Bond Finance and Certification. BIS Quarterly Review Special Features Series. 2017.

[32] Larcker FD, Watts ME. Where’s the greenium? Journal of Accounting and Economics. 2020; 69(2–3):101312. doi: 10.1016/j.jacceco.2020.101312

[33] Malcolm B, Daniel B, George S, Jeffrey W. The pricing and ownership of US green bonds. Annual Review of Financial Economics. 2022; 14:415–437. doi: 10.1146/ANNUREV-FINANCIAL-111620-014802

[34] Sun HM, Lei YJ. Research on cost difference and influencing factors of ‘green financing’—Based on analysis of green bond credit spread. Finance Forum. 2023; 28(02):34–45. doi: 10.16529/j.cnki.11-4613/f.2023.02.008

[35] Andersson S, Prag K. Green bonds: Doing well by doing good. Lund University; 2015.

[36] Karpf A, Mandel A. The changing value of the ‘green’ label on the US municipal bond market. Nature Climate Change. 2018; 8(2):161–165 doi: 10.1038/s41558-017-0062-0

[37] Febi W, Schäfer D, Stephan A, Chen S. The impact of liquidity risk on the yield spread of green bonds. Finance Research Letters. 2018; 27:53–59. doi: 10.1016/j.frl.2018.02.025

[38] Eichholtz P, Holtermans R, Kok N, Yönder E. Environmental performance and the cost of debt: Evidence from commercial mortgages and REIT bonds. Journal of Banking and Finance. 2019; 102:19–32. doi: 10.1016/j.jbankfin.2019.02.015

[39] Antonio D, Ana E. Sustainability premium in energy bonds. Energy Economics. 2021; 95(prepublish):105113-. doi: 10.1016/J.ENECO.2021.105113

[40] Jun MA. On the construction of China’s green finance system. Finance Forum. 2015; 20(05):18–27. doi: 10.16529/j.cnki.11-4613/f.2015.05.002

[41] Wang SH, Lin XY, Zhang W, Li QY. Research on the impact of green credit on green technology innovation efficiency of industry in China. Journal of Statistics and Information. 2023; 38(04):88–102.

[42] Ning JH, Wang M. Can green bonds ease the maturity of investment and financing of enterprises? Empirical evidence from the bond market. Securities Market Herald. 2021; (09):48–59.

[43] Clifford W, SMITH J. Investment banking and the capital acquisition process. Journal of Financial Economics. 1986; 15(1–2):3–29. doi: 10.1016/0304-405X(86)90048-6

[44] Liu EP. Convertible bond issuance announcement of Chinese listed companies. Journal of Financial Research. 2005; (07):45–56.

[45] Fu LM, Wan DF, Zhang YH. An empirical study on the announcement effect of corporate bond issuance of Chinese listed companies. Journal of Financial Research. 2010; (03):130–143.

[46] Castillo A. The announcement effect of bond and equity issues: evidence from Chile. University of Chile, Department of Economics University of Chile, Department of Economics. 2004; 31(2):177–205.

[47] Roslen MNS, Yee SL, Ibrahim BAS. Green bond and shareholders’ wealth: a multi-country event study. International Journal of Globalisation and Small Business. 2017; 9(1):61–69. doi: 10.1504/IJGSB.2017.084701

[48] Chen DN. The stock price effect of green bond issuance of Chinese listed companies. Journal of Shanxi Finance and Economics University. 2018; 40(S2):35–38.

[49] Liang ZH. Research on the market reaction of green bond issuance and the green preference of stock investors. Journal of Regional Financial Research. 2018; (09):44–48.

[50] Chen FG, Zhang YH. Can green bond issuance trigger a benign market reaction?—Based on the policy incentive effect of the ’ double carbon ’ goal. Securities Market Herald. 2022; (07):48–60.

[51] Qi HJ, Liu SQ. Is there a greenium in the bond market of China? Accounting Research. 2021; (11):131–148.

[52] Wu YH, Tian YL, Chen YY, Xu Q. The spillover effect, mechanism and performance of green bond issuance. Management World. 2022; 38(06):176–193. doi: 10.19744/j.cnki.11-1235/f.2022.0086

[53] Liu CZ, Ren Y. Research on the impact of green credit on the low carbonization of energy consumption structure. Wuhan Finance. 2019; (11):66–70.

[54] Wen ZL, Fang J, Xie JY, Ouyang JY. Methodological research on mediation effects in China’s mainland. Advances in Psychological Science. 2022; 30(08):1692–1702.

