# Correction: The impact of green bond issuance on carbon emission intensity and path analysis

**DOI:** 10.1371/journal.pone.0312399

**Published:** 2024-10-15

**Authors:** Haifeng Pang, Changxu Wu, Liucheng Zhang

There is an error in affiliation 1 for the authors Haifeng Pang, Changxu Wu and Liucheng Zhang. The correct affiliation 1 is: Harbin University of Commerce, Harbin, Heilongjiang Province, China
