# Supplementary material for: The impact of green bond issuance on carbon emission intensity and path analysis
Source: PLoS One. 2024 Jun 5;19(6):e0304364. doi: 10.1371/journal.pone.0304364 (PMC11152263; doi:10.1371/journal.pone.0304364)
Supplement: S1 Appendix — (DOCX) [file pone.0304364.s002.docx]

**S1 Appendix**

Panel-corrected standard error estimation test results

|  | Value | P-Value |  |
| --- | --- | --- | --- |
| Greenbonds | $\mathop{-0.106^{**}}\limits_{(-2.15)}$ | 0.031 |  |
| _cons | $\mathop{9.805}\limits_{(1.51)}$ | 0.131 |  |
| Heteroskedasticity | 400.61 | 0.0000 |  |
| Autocorrelation | 56.234 | 0.0000 |  |
| Note:  indicate significance at 1%, 5%, and 10% significance levels, respectively, with t-statistics in parentheses. | | |  |
