# Supplementary material for: The impact of green bond issuance on carbon emission intensity and path analysis
Source: PLoS One. 2024 Jun 5;19(6):e0304364. doi: 10.1371/journal.pone.0304364 (PMC11152263; doi:10.1371/journal.pone.0304364)
Supplement: S2 Appendix — (DOCX) [file pone.0304364.s003.docx]

**S2 Appendix**

Model lagged multi-period regression results

|  | One-phase lag | | Two-phase lag | | Three-phase lag | |
| --- | --- | --- | --- | --- | --- | --- |
|  | $W_{1}$ | $W_{2}$ | $W_{1}$ | $W_{2}$ | $W_{1}$ | $W_{2}$ |
| lCi_1 | $\mathop{0.515^{***}}\limits_{(11.49)}$ | $\mathop{0.510^{***}}\limits_{(8.04)}$ | $\mathop{0.690^{***}}\limits_{(6.10)}$ | $\mathop{0.718^{***}}\limits_{(5.20)}$ | $\mathop{0.783^{***}}\limits_{(8.11)}$ | $\mathop{0.783^{***}}\limits_{(8.93)}$ |
| lCi_2 |  |  | $\mathop{-0.158^{*}}\limits_{(-1.92)}$ | $\mathop{-0.182^{*}}\limits_{(-1.82)}$ | $\mathop{-0.238^{**}}\limits_{(-1.59)}$ | $\mathop{-0.403^{**}}\limits_{(-2.19)}$ |
| lCi_3 |  |  |  |  | $\mathop{-0.008}\limits_{(-0.05)}$ | $\mathop{0.150}\limits_{(0.82)}$ |
| Greenbonds | $\mathop{-0.009}\limits_{(-0.84)}$ | $\mathop{-0.013^{*}}\limits_{(-1.17)}$ | $\mathop{-0.009}\limits_{(-1.08)}$ | $\mathop{-0.011}\limits_{(-1.25)}$ | $\mathop{0.007}\limits_{(0.81)}$ | $\mathop{-0.012}\limits_{(-1.64)}$ |
| W×Greenbonds | $\mathop{-0.004}\limits_{(-0.29)}$ | $\mathop{0.044}\limits_{(1.05)}$ | $\mathop{-0.003}\limits_{(-0.20)}$ | $\mathop{0.049}\limits_{(0.99)}$ | $\mathop{0.005}\limits_{(0.30)}$ | $\mathop{-0.022}\limits_{(-0.42)}$ |
| Rho | $\mathop{0.212^{***}}\limits_{(2.66)}$ | $\mathop{0.227}\limits_{(2.07)}$ | $\mathop{0.220^{***}}\limits_{(2.78)}$ | $\mathop{0.233}\limits_{(0.02)}$ | $\mathop{0.181^{***}}\limits_{(2.57)}$ | $\mathop{0.122}\limits_{(0.94)}$ |
| Year | Yes | Yes | Yes | Yes | Yes | Yes |
| N | 156 | 156 | 156 | 156 | 156 | 156 |
| $R^{2}$ | 0.7731 | 0.7795 | 0.7680 | 0.7505 | 0.7969 | 0.7678 |
| Note:  indicate significance at 1%, 5%, and 10% significance levels, respectively, with t-statistics in parentheses. | | | | | | |
